# Co‐Micronized Palmitoylethanolamide and Rutin Associated With Hydroxytyrosol Recover Diabesity‐Induced Hepatic Dysfunction in Mice: In Vitro Insights Into the Synergistic Effect

**DOI:** 10.1002/ptr.8361

**Published:** 2024-10-30

**Authors:** S. Melini, C. Pirozzi, A. Lama, F. Comella, N. Opallo, F. Del Piano, E. Di Napoli, M. P. Mollica, O. Paciello, M. C. Ferrante, G. Mattace Raso, R. Meli

**Affiliations:** ^1^ Department of Pharmacy, School of Medicine University of Naples Federico II Naples Italy; ^2^ Department of Veterinary Medicine and Animal Productions University of Naples Federico II Naples Italy; ^3^ Department of Biology University of Naples Federico II Naples Italy

**Keywords:** diabetes, flavonoids, MAFLD, N‐acylethanolamine, obesity, polyphenols

## Abstract

Metabolic dysfunction‐associated fatty liver disease (MAFLD) and diabesity (diabetes related to obesity) are interrelated since glucose and lipid alterations play a vital role in the development of both disorders. Due to their multi‐variant metabolic features, more than one drug or natural product may be required to achieve proper therapeutic effects. This study aimed to evaluate the effectiveness of a formulation containing co‐micronized palmitoylethanolamide and rutin (PEA‐Rut) associated with hydroxytyrosol (HT), namely NORM3, against hepatic damage and metabolic alterations in high‐fat diet (HFD)‐induced diabesity in mice. NORM3 decreased the body weight and fat mass of obese mice. The formulation improved HFD‐altered insulin sensitivity and hepatic glucose production and metabolism, as shown by glucose, insulin, pyruvate tolerance tests, Western blot, and real‐time PCR. In the liver, NORM3 limited macro‐ and micro‐vacuolar steatosis, as revealed by morphological analysis, and reduced the associated hepatic inflammation. NORM3 counteracted lipid dysfunctions of HFD animals, activating AMPK, a key cellular energy sensor, and normalizing the expression of carnitine palmitoyl‐transferase (CPT)1, a rate‐limiting enzyme of fatty acid β‐oxidation, and other genes involved in lipid homeostasis. Relevantly, the hepatic antioxidant activity of NORM3 was proved (reduced ROS and increased detoxifying factors and enzymes). Finally, in vitro synergistic protective effects of the components (PEA‐Rut and HT) on H_2_O_2_‐induced oxidative challenge in HepG2 were determined (ROS production, inflammation, and antioxidant defense). Our results show the beneficial effect of NORM3 and its potential as an innovative phytotherapeutic combination in limiting hepatic damage progression and counteracting glucose and lipid dysmetabolism associated with diabesity.

## Graphical abstract

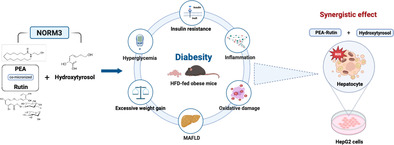

**Abbreviations**
ALP: alkaline phosphatase
ALT: alanine aminotransferase
AST: aspartate aminotransferase
AUC: area under the curve
CD: cluster of differentiation
COX: cyclooxygenase
CPT: carnitine palmitoyl‐transferase
DMSO: dimethyl sulfoxide
FFAs: free fatty acids
H&E: hematoxylin & eosin
HFD: high‐fat diet
HO‐1: heme oxygenase‐1
HOMA: homeostasis model assessment
HT: hydroxytyrosol
IFN: interferon
IL: interleukin
InsR: insulin receptor
IR: insulin resistance
ITT: insulin tolerance test
LDH: lactate dehydrogenase
MAFLD: metabolic‐associated fatty liver disease
MCP: monocyte chemoattractant protein
NAE: N‐acylethanolamine
NAFLD: non‐alcoholic fatty liver disease
NASH: steatohepatitis
NORM3: NORMAST 3
NOX: NADPH oxidase
NRF‐2: nuclear factor erythroid 2‐related factor 2
OGTT: oral glucose tolerance test
PBS: phosphate buffer solution
PEA: palmitoylethanolamide
PEA‐Rut: palmitoylethanolamide‐rutin
PPAR: peroxisome proliferator‐activated receptor
PTT: pyruvate tolerance test
TNF: tumor necrosis factor

## 1 Introduction

Over the past decade, diabesity, namely diabetes related to obesity, has been a term used to underline the pathological interconnections of these dual epidemics and detrimental disorders (Michaelidou, Pappachan, and Jeeyavudeen 2023). It is well known that up to 85.2% of type 2 diabetes patients are overweight or obese (Ruze et al. 2023). Along with the epidemic of diabesity, the global prevalence of non‐alcoholic fatty liver disease (NAFLD) is growing (Younossi et al. 2019). NAFLD and diabesity are epidemiologically interrelated, and their causal relationship has been determined (Liu et al. 2020).

Since NAFLD is closely related to several metabolic diseases, the term metabolic‐associated fatty liver disease (MAFLD) was proposed in 2020 to point out better the shared etiology of the concurrent conditions associated with hepatic dysmetabolism (Eslam et al. 2020; Nan et al. 2021). MAFLD is defined based on evidence of hepatic steatosis and its coexistence with at least one of the following conditions: obesity, diabetes, or metabolic dysregulation (Eslam et al. 2020). A recent study used a comprehensive meta‐analysis to report the worldwide incidence and prevalence of type 2 diabetes among individuals with NAFLD or MAFLD (Cao et al. 2024).

The liver is the maintenance hub of glucose and lipid homeostasis. A variation in liver metabolic function occurs in response to the alteration of this homeostasis and hepatic insulin resistance (IR). IR stimulates hepatic excessive lipid storage, which in turn can aggravate IR in a detrimental vicious loop (Chen et al. 2017) and contributes to the so‐called gluco‐ and lipo‐toxicity (Mota et al. 2016; van der Zande et al. 2021). In this context, chronic low‐grade inflammation or meta‐inflammation is one of the main contributors to liver‐specific IR and impaired glucose/lipid homeostasis and oxidative damage (Luo and Lin 2021; Rohm et al. 2022). Restoring insulin sensitivity, glucose synthesis, and storage in the liver could counteract glucose dysmetabolism in diabesity (Lopez‐Soldado, Guinovart, and Duran 2023; Lopez‐Soldado et al. 2015). In this regard, many lines of evidence are moving toward the nuclear peroxisome proliferator‐activated receptor (PPAR) modulators (Annunziata et al. 2020; Pirozzi et al. 2023), as well as antioxidant natural compounds and functional food (Lama et al. 2017; Pirozzi et al. 2016) with broad‐spectrum pharmacological activity and minimal risk of side effects (Li et al. 2021; Rudrapal et al. 2022).

Palmitoylethanolamide (PEA) is a bioactive endogenous lipid belonging to the N‐acylethanolamine (NAE) family synthesized “on demand” from membrane phospholipids. It exerts a plethora of biological effects, involved in inflammation, pain, and metabolism control, which are mainly due to its interaction with the PPAR‐α (Mattace Raso et al. 2014; Russo et al. 2018). In hepatocytes, PPAR‐α regulates the expression of genes involved in lipid metabolic pathways, controlling trafficking, delivery, and storage of free fatty acids (FFAs) or enzyme function involved in mitochondrial β‐oxidation (Kersten 2014; Lin, Wang, and Li 2022). Hepatic FFA accumulation also derives from triglyceride mobilization from adipose tissue. It is associated with a decrease in PPAR‐α activation, contributing to lipotoxicity in steatohepatitis (NASH) due to NAFLD progression (Todisco et al. 2022).

Polyphenolic compounds, naturally found in plant foods (fruits, vegetables, and medicinal plants), benefit human health and protect against chronic non‐communicable diseases, such as cardiovascular diseases and obesity. Experimental studies showed that plant‐based foods and their bioactive compounds had first‐rate protective properties in steatosis, oxidative stress, inflammation, and gut dysbiosis related to NAFLD progression (Li et al. 2021).

Among all, hydroxytyrosol (HT), the major metabolite of oleuropein, found in the aqueous fraction of extra virgin olive oil, and the flavonoid rutin, contained in many typical plants (buckwheat, passionflower, apple, and tea) plays an essential role in the hepatic detoxification of free radicals (de Pablos et al. 2019; Mosca et al. 2021; Pirozzi et al. 2016).

Since diabesity is a multi‐variant metabolic disorder, a single drug or natural product may be insufficient to show the desired therapeutic effect. Thus, identifying the combination of safe and useful natural active products could provide an appealing approach to counteract this multifactorial disorder and prevent hepatic disease progression. Consistently, the dietary supplement NORMAST 3 (NORM3), a patent formulation containing co‐micronized PEA and rutin associated with HT (as diabetes relievers), may offer an effective and safe intervention for diabesity, limiting MAFLD and its progression.

Here, we report that NORM3 dampens the hepatic dysmetabolism, reducing inflammation and oxidative stress, in an experimental mouse model of diabesity and related comorbidities induced by high‐fat feeding. The in vitro synergistic effect of the mixture components was also proved.

## 2 Material and Methods

### 2.1 Preparation of NORMAST 3

Palmitoylethanolamide‐Rutin (PEA‐Rut) 5:1 homogeneously premixed powder was micronized by standard air jet‐mill technology (Remington's Pharmaceutical Sciences Handbook, Mack Pub. Co., NY, USA, 22nd edition). Regular and reproducible particle size > 90% in the 2.0–10‐μm range was achieved. The micronized powder was mixed with 20% HT to reach the final w/w ratio of NORM3 that is 10:2:0.5.

### 2.2 Animals and In Vivo Procedures

Young male C57Bl/6J mice (6 weeks of age, Charles River, Wilmington, MA, USA) were housed in stainless steel cages at 22°C ± 1°C with a 12:12 h light–dark cycle. Animals were fed with a standard chow diet (STD, *n* = 11) containing 17% of energy derived by fat, without sucrose (4RF18, Mucedola srl, Milan, Italy), or a high‐fat diet (HFD, *n* = 22) (D12451, Research Diets Inc., NJ, USA) that had 45% of energy derived from fat and 7% of sucrose. After 12 weeks, diabesity was established by evaluating (i) the significant weight gain (STD = 30.19 g ± 0.24 and HFD = 42.85 g ± 1.04, ****p < 0.0001 vs. STD) and (ii) the fasting hyperglycemia of HFD animals compared with STD group (STD = 110.2 mg/dL ± 8.49 and HFD = 164.3 mg/dL ± 9.33, ***p < 0.001 vs. STD). Then, HFD mice were divided into two subgroups (*n* = 11 for each group): HFD mice receiving vehicle, and HFD animals treated with NORM3 (PEA 10 mg/kg/die‐rutin 2 mg/kg/die and HT 0.5 mg/kg/die per os) from week 12 up to week 19. Co‐micronized PEA‐Rut and HT (synthesized and provided by Epitech group S.p.A Padova, Italy) were suspended in carboxymethyl cellulose (1.5%). All animals were analyzed for body weight determination. Then, we used different sets of animals belonging to the same experimental groups for different biochemical and molecular evaluations, as well as fat mass determination. Given the distress of fat mass measurement due to anesthetic procedures, we performed this determination on fewer animals, which can allow us to reach the possible significant difference among groups. At the end of the 7th week of NORM3 treatment, the fat mass was evaluated by bioelectrical impedance analysis (BIA) as previously described (Rutter et al. 1998) and then modified for mice by Mollica et al. (2017). Briefly, a weak electric current flows through the mouse body, and the voltage is measured to calculate the body's impedance (resistance and reactance). Then, the animals were anesthetized with inhaled isoflurane (cat. number B506, Abbott S.r.l., Rome, Italy) followed by intracardiac puncture and cervical dislocation, and serum and tissues were collected, frozen, and stored at −80°C.

### 2.3 Ethics Statement

All procedures concerning animals and their care were carried out according to national and international law and policies (Italian D.L. No. 26 of March 4, 2014, and EU Directive 2010/63/EU for animal experiments, ARRIVE guidelines and the Basel declaration, including the 3R concept). The procedures reported here were approved by the Institutional Committee on the Ethics of Animal Experiments (CSV) of the University of Naples Federico II and by the Italian Ministry of Health under protocol no. 140/2022‐PR.

### 2.4 Glucose, Insulin, and Pyruvate Tolerance Tests

At the end of the experimental period, oral glucose, insulin, and pyruvate tolerance tests (OGTT, ITT, and PTT) were performed (at least six animals for each group). For OGTT and PTT, mice were fasted for 16 h, while for ITT, mice starved for 6 h.

Briefly, after fasting blood glucose measurement, animals received an aqueous solution of glucose (1 g/kg, cat. number G8270, Sigma–Aldrich, Milan, Italy) by oral gavage or intraperitoneal injection of pyruvate (2 g/kg, cat. number P2256, Sigma–Aldrich, Milan, Italy) or insulin (0.75 IU/kg, Humulin R, cat. number HI210, Ely Lilly, Indianapolis, IN, USA). Glucose levels were measured in tail vein blood at different selected time points (0, 30, 60, 90, and 120 min for OGTT and 0, 15, 30, 60, and 120 min for ITT and PTT) using a blood glucometer (One Touch Verio). The area under the curve (AUC) was calculated as an integrated and cumulative measurement of glycemia from time 0 up to 120 min for each animal.

### 2.5 Biochemical, Hormone, and Hepatic Determinations

At the end of the experimental time, blood was collected by intracardiac puncture from all experimental groups of mice. Serum samples were obtained by centrifugation at 2500 rpm at 4°C for 12 min and then stored at 80°C. Serum insulin, leptin, ghrelin, resistin, PAI‐1, glucagon, GLP1, and GIP concentrations were measured using Bio‐Plex Pro Mouse Diabetes 8‐Plex Assay kit (cat. number 171F7001M, Bio‐Rad Laboratories, Milan, Italy) according to the manufacturer's instructions. Furthermore, serum alanine aminotransferase (ALT), aspartate aminotransferase (AST), lactate dehydrogenase (LDH), cholesterol, triglycerides, adiponectin (cat. numbers AL146, AS101, LD3842, CH200, TR210, respectively, Randox Laboratories ltd., Crumlin, UK), and pro‐inflammatory cytokines as interleukin (IL)‐1β, IL‐6, IL‐10, tumor necrosis factor (TNF)‐α, monocyte chemoattractant protein (MCP)‐1, and interferon (IFN)‐γ levels (cat. numbers MLB00C, M100B, M6000B, MTA00B, MJE00B, and MIF00, respectively, R&D Systems, Inc., Minneapolis, MN) were measured by colorimetric enzymatic method using commercially available ELISA kits. Then, we calculated the Homeostasis Model Assessment (HOMA‐IR) using the formula (HOMA = fasting glucose [mmol/L] × fasting insulin [μU/mL]/22.5).

### 2.6 Liver Histological Evaluations

Livers were removed, weighed, fixed in neutered buffered formalin, and embedded in paraffin. Ten‐micrometer sections were stained with hematoxylin & eosin (H&E) for morphological assessment. Livers were subjected to blinded histomorphometric analysis and analyzed by the same pathologist to perform a semi‐quantitative evaluation. Steatosis was scored on a scale of 0 (no steatosis), 1 (mild, 1 < 30% of affected hepatocytes), 2 (moderate, 30%–70% of affected hepatocytes), and 3 (severe, > 70% of affected hepatocytes).

### 2.7 Cell Culture and Treatments

Human HepG2 cells (American Type Culture Collection, Manassas, VA) were cultured in RPMI 1640 complete medium (cat. number 11875093, Gibco Thermo Fisher Scientific, USA) at 37°C with 5% CO_2_. Cells were seeded in P6‐well plates (5.0 × 10^5^ cells/well) and starved for 6 h in 5% FBS medium. Cells were pre‐treated with PEA‐Rut (1 μM) and HT (50 nM) alone or in combination in the same ratio between the components contained in NORM3, as well as their vehicle for 6 h, before H_2_O_2_ (30% cat. number H1009, Sigma–Aldrich, Milan, Italy) challenge (400 μM for 4 h) to obtain oxidative and inflammatory damage.

### 2.8 ROS Production

ROS assay was performed as previously reported (Pirozzi et al. 2020) in both liver and HepG2 cells. To quantify ROS production, an appropriate volume of freshly prepared liver homogenate was diluted in 100 mM potassium phosphate buffer (pH 7.4) and incubated with 5 μM dichloro‐fluorescein diacetate (cat. number 35845, Sigma–Aldrich, Milan, Italy) in dimethyl sulfoxide (DMSO, cat. number D5879, Sigma–Aldrich, Milan, Italy) for 15 min at 37°C. The dye‐loaded samples were centrifuged at 12,500×*g* per 10 min at 4°C, and then 5 mL of 100 mM potassium phosphate buffer (pH 7.4) was added to the pellet and incubated for 60 min at 37°C. The fluorescence was measured using the Promega GloMax Explorer Microplate Reader (cat. number GM3560, Milan, Italy) at 488 nm for excitation and 525 nm for emission wavelengths. ROS were quantified from the dichloro‐fluorescein standard curve in DMSO (0–1 mM).

### 2.9 Western Blot Analysis

Total protein lysates, obtained by liver homogenization, were subjected to SDS–PAGE as previously described (Pirozzi et al. 2023). The blot was performed by Trans‐Blot Turbo transfer system (cat. number 704150, Bio‐Rad Laboratories, Segrate, Milan, Italy) at room temperature for 240 mA for 60 min. The obtained membrane filter was then blocked with 1× phosphate buffer solution (PBS) and 5% nonfat dried milk for 60 min at room temperature and probed with anti‐phospho‐insulin receptor (InsR)β mouse polyclonal antibody (dilution 1:1000, cat. number 44809G, Thermo Fisher Scientific, cat. number: 32109, Rodano (MI), Italy), anti‐InsRβ rabbit monoclonal antibody (dilution 1:1000, cat. number 3025), anti‐phosphatidylinositol 3 kinase (PI3K) rabbit monoclonal antibody (dilution 1:1000, cat. number 4249S), anti‐phospho‐AKT (Ser473) rabbit monoclonal antibody (dilution 1:1000, cat. number 4060), anti‐AKT (pan) mouse monoclonal antibody (dilution 1:1000, cat. number 4691), anti‐phospho‐AMPKα rabbit monoclonal antibody (dilution 1:1000, cat. number 2535), and anti‐AMPKα mouse monoclonal antibody (dilution 1:1000, cat. number 2793) (Cell Signaling Technology, Inc., Beverly, MA, USA), anti‐CPT1L rabbit polyclonal antibody (dilution 1:500, cat. number A30939, Bioworld Technology, Inc), anti‐cyclooxygenase (COX)‐2 mouse monoclonal antibody (dilution 1:200, cat. number), anti‐NFκB p65 subunit rabbit monoclonal antibody (dilution 1:200, cat. number sc8008) (Santa Cruz Biotechnology, Inc., USA). β‐Actin mouse polyclonal antibody (dilution 1:2000, cat. number A5441, Sigma–Aldrich, Milan, Italy) was used as housekeeping to ensure equal sample loading. The signals were visualized with the ECL system (Pierce, Thermo Fisher Scientific, cat. number 32109, Rodano (MI), Italy) for chemiluminescence assay (ChemiDoc Imaging Systems, cat. number 12003153, Bio‐Rad Laboratories, Segrate, Milan, Italy). Uncropped images for all blots were reported in Figure S1.

### 2.10 Semi‐Quantitative Real‐Time PCR Analysis

Total RNA isolated from the liver or HepG2 cells was extracted using PureZOL RNA Isolation Reagent (cat. number: 7326890, Bio‐Rad Laboratories, Segrate, Milan, Italy) following the extraction kit's protocol for RNA (cat. number FC140955N, NucleoSpin, Macherey‐Nagel GmbH & Co, Düren, Germany). cDNA was obtained using a High‐Capacity cDNA Reverse Transcription Kit (cat. number 4374966, Applied Biosystems, Foster City, CA, USA) from 4 to 8 μg total RNA. RT‐PCRs were performed with a Bio‐Rad CFX96 Connect Real‐time PCR System instrument and software (Bio‐Rad Laboratories, Milan, Italy). The RT‐PCR conditions were previously described (Lama et al. 2021). Each sample contained 500 ng cDNA in 2X QuantiTect SYBR Green PCR Master Mix (cat. number 204145, Qiagen, Hilden, Germany) and primers pairs to amplify phospho‐enol pyruvate carboxy kinase (*Pck1*, QT00153013), glucose‐6‐phosphatase (*G6pc*, QT00114625), PPAR‐α (*Ppara*, QT00137984), cluster of differentiation (CD)36 (*Cd36*, cat. number), fatty acid synthase (*Fasn*, QT00149290), IL‐1β (*Il1b*, mouse QT010485355, human QT00021385), TNF‐α (*Tnf*, QT00104006), nuclear factor erythroid 2‐related factor 2 (NRF‐2 or *Nfe2l2*, mouse QT00095270, human QT00027384), NAD(P)H quinone dehydrogenase 1 (*Nqo1*, QT00094367), and heme oxygenase‐1 (HO‐1 or *Hmox1*, QT00159915) (Qiagen, Hilden, Germany), in a final volume of 100 μL. The relative amount of each studied mRNA was normalized to GAPDH (*Gapdh*, mouse QT01658692, human QT00079247) (Qiagen, Hilden, Germany) as a housekeeping gene, and data were analyzed according to the 2^−∆∆Ct^ method.

### 2.11 Statistical Analysis

All data shown are presented as mean value ± S.E.M. Shapiro–Wilk normality test was performed on all analyzed data. Statistical analysis was performed by one‐way or two‐way ANOVA (minimum *n* = 6 animals in each group), depending on different analyses, followed by Bonferroni post hoc for multiple comparisons. Differences among groups were considered significant at values of *p* < 0.05. Analyses were performed using GraphPad Prism 9 (GraphPad Software, San Diego, CA, USA).

## 3 Results

### 3.1 Effect of NORM3 on Body Weight and Glucose Homeostasis of Obese Mice

**FIGURE 1:**
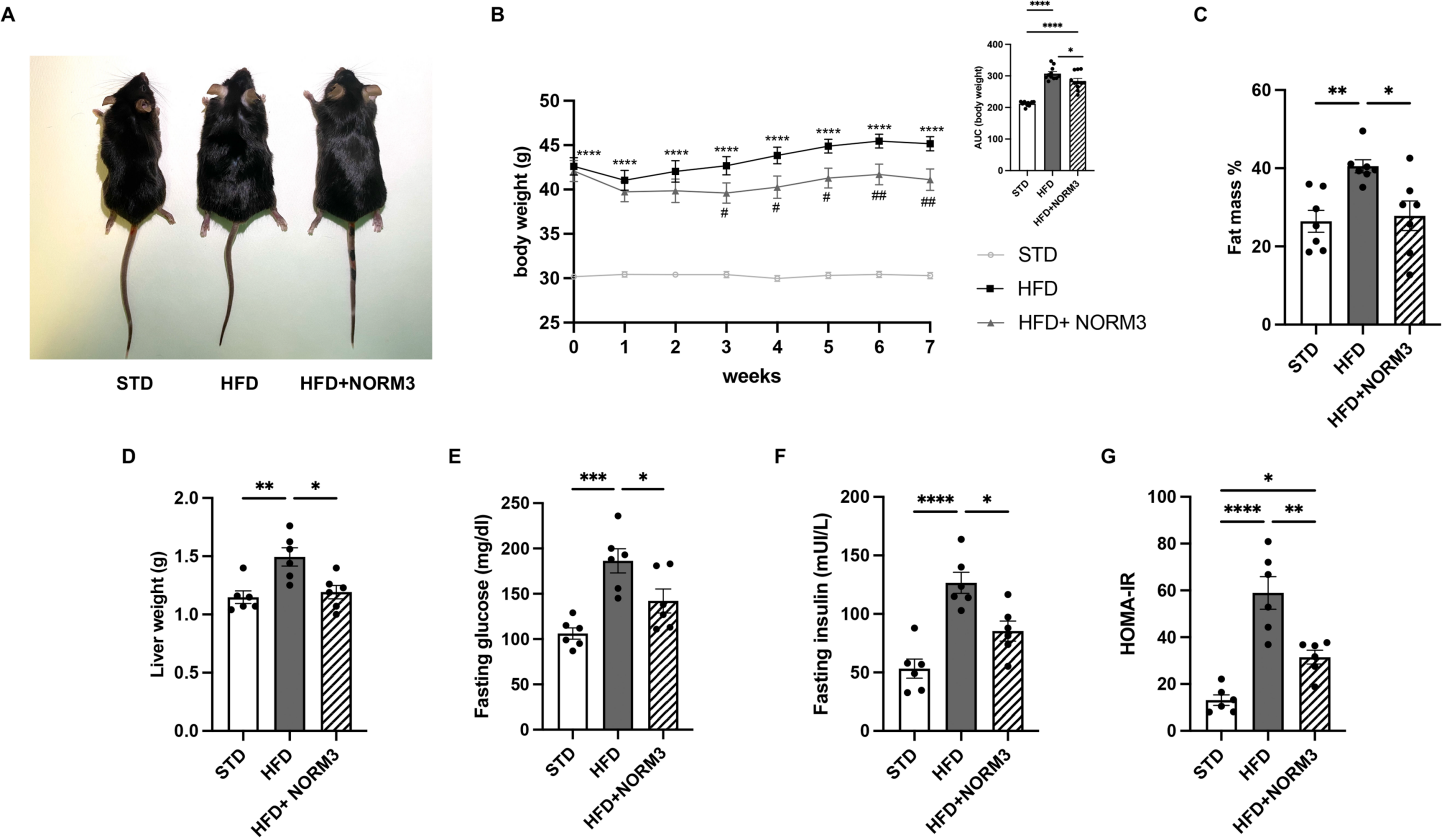
NORM3 reduces body weight and metabolic parameters altered in HFD‐induced obesity. Macroscopic representation of NORM3 effect on obese phenotype in mice (A); body weight and respective AUC were measured throughout the experimental period (*n* = 11 each group) (B). Fat mass was assessed by bioelectrical impedance analysis at the end of 7th week of treatment (*n* = 7 each group) (C), liver weight (D), fasting glucose (E), insulin (F), and HOMA‐IR (G) were determined after sacrifice (*n* = 6 each group). Data for XY graphs are presented as mean ± SEM. ****p < 0.0001 versus STD; ^#^ *p* < 0.05, and ^##^ *p* < 0.01 versus HFD. Data for column bar graphs are presented as mean ± SEM. *p < 0.05, **p < 0.01, ***p < 0.001, and ****p < 0.0001 versus STD or HFD.

**FIGURE 2:**
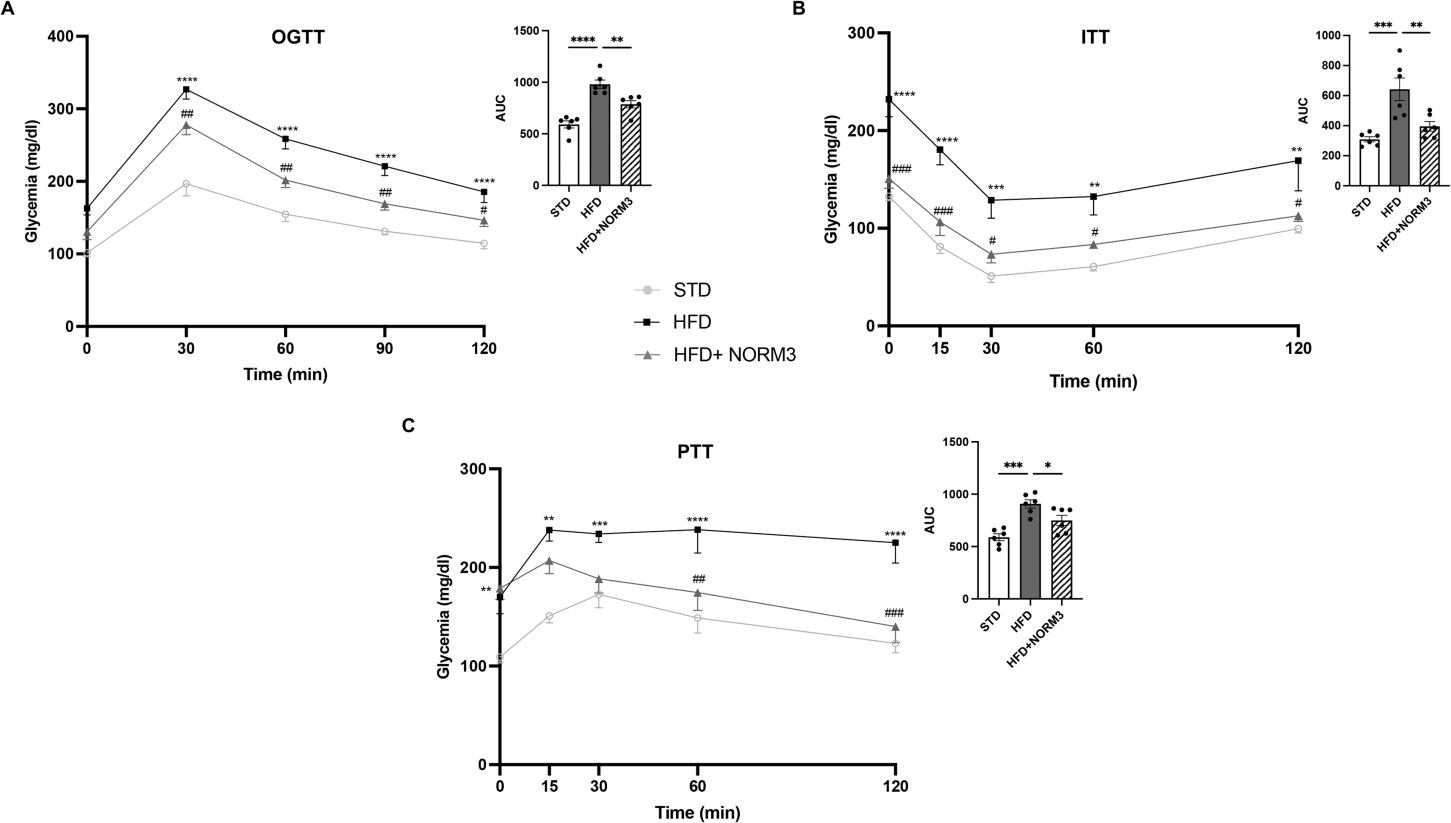
Effect of NORM3 on glucose homeostasis compromised by HFD feeding. Oral glucose tolerance test (OGTT) (A) and intraperitoneal insulin (B) and pyruvate (C) tolerance tests (ITT and PTT) and respective AUC were shown for different sets of animals from all groups of mice (*n* = 6 each group). Data for XY graphs are presented as mean ± SEM. **p < 0.01, ***p < 0.001, and ****p < 0.0001 versus STD; ^#^ *p* < 0.05, ^##^ *p* < 0.01, ^###^ *p* < 0.001 versus HFD. Data for column bar graphs are presented as mean ± SEM. *p < 0.05, **p < 0.01, ***p < 0.001, and ****p < 0.0001 versus STD or HFD.

NORM3 administration macroscopically attenuated obese phenotype in mice (Figure 1A). It reduced the body weight compared with the untreated HFD group starting from the 3rd week (Figure 1B), as confirmed by the area under the curve of the body weight measured up to the 7th week (AUC, upper panel). Consistently, NORM3 treatment reduced fat mass and liver weight, which significantly increased by HFD feeding (Figure 1C,D). NORM3 lessened IR, reducing glucose and insulin and improving HOMA‐IR (Figure 1E–G). Furthermore, NORM3 improved blood glucose homeostasis and insulin sensitivity as assessed by OGTT and ITT (Figure 2A,B). In particular, NORM3 limited the alteration of glycemia at each analyzed time point and the relative AUC, as shown in the respective upper panels. The lowering effect of glycemia by NORM3 was also observed in HFD‐fed animals during PTT (Figure 2C), suggesting its effectiveness in reducing gluconeogenesis.

### 3.2 Effect of NORM3 Treatment on Serum Biochemical and Hormonal Profile

The evaluation of biochemical markers of liver function and serum inflammation showed a protective effect of NORM3 in obese mice (Table 1). Indeed, NORM3 reduced HFD‐altered systemic levels of ALT, AST, ALP, triglycerides, cholesterol, LDH, and pro‐inflammatory mediators TNF‐α, IL‐6, and MCP‐1 and increased the anti‐inflammatory IL‐10. Moreover, NORM3 restored serum glucagon and resistin to STD levels and induced a reduction trend of PAI‐1, a physiological inhibitor of plasminogen activators, compared with untreated HFD, all factors involved in IR occurring in diabesity. Other serum hormones, including GIP, GLP1, and ghrelin, did not significantly change among groups (Table 2).

**TABLE 1:** Effect of NORM3 on serum hepatic and inflammatory parameters in HFD‐fed mice.

Serum parameters	STD	HFD	HFD + NORM3
ALT (U/L)	64.17 ± 3.79	131.40 ± 6.37^****^	108.30 ± 5.41^#^ ^,^ ^****^
AST (U/L)	63.83 ± 5.25	117.40 ± 5.63^****^	86.33 ± 2.75^##^ ^,^ **
ALP (U/L)	150.20 ± 4.93	265.60 ± 4.33^****^	197.00 ± 3.65^####^ ^,^ ^****^
Triglycerides (mg/dL)	110.00 ± 3.29	216.80 ± 5.05^****^	173.20 ± 5.42^####^ ^,^ ^****^
Cholesterol (mg/dL)	136.80 ± 3.36	259.20 ± 5.68^****^	190.70 ± 6.39^####^ ^,^ ^****^
LDH (U/L)	68.33 ± 3.13	134.20 ± 7.40^****^	82.20 ± 2.75^####^ ^,^ ***
TNF‐α (ng/mL)	1.04 ± 0,06	3.67 ± 0.15^****^	1.53 ± 0.09^####^ ^,^ *
IL‐6 (pg/mL)	22.00 ± 0.09	42.60 ± 4.14^****^	26.17 ± 0.94^###^
MCP‐1 (pg/mL)	29.83 ± 1.17	64.20 ± 1.93^****^	39.00 ± 1.88^####^ ^,^ **
IL‐10 (ng/mL)	0.03 ± 0.01	0.03 ± 0.01	0.05 ± 0.01^##^

*Note*: Data are presented as mean ± SEM of all animals from different groups (*n* = 5–6 for each group).

* *p* < 0.05.

** *p* < 0.01.

*** *p* < 0.001.

**** *p* < 0.0001 versus STD.

^#^ *p* < 0.05.

^##^ *p* < 0.01.

^###^ *p* < 0.001.

^####^ *p* < 0.0001 versus HFD.

**TABLE 2:** Effect of NORM3 treatment on serum hormonal profile of obese animals.

Serum parameters	STD	HFD	HFD + NORM3
Glucagon (ng/mL)	0,51 ± 0.04	0,72 ± 0.03**	0,55 ± 0,03^#^
Resistin (ng/mL)	52.26 ± 4.79	99.02 ± 11.24**	61.75 ± 4.63^##^
PAI‐1 (ng/mL)	1.16 ± 0.11	3.23 ± 0.64**	1.85 ± 0.18
GIP (ng/mL)	3.10 ± 0.16	2.70 ± 0.32	3.48 ± 0.27
GLP‐1 (ng/mL)	0.08 ± 0.02	0.06 ± 0.01	0,09 ± 0.01
Ghrelin (ng/mL)	1,88 ± 0,24	3,59 ± 0,68	3,70 ± 0,65

*Note*: Data are presented as mean ± SEM of all animals from different groups (*n* = 6 for each group).

** *p* < 0.01 versus STD.

^#^ *p* < 0.05.

^##^ *p* < 0.01 versus HFD.

### 3.3 Effect of NORM3 on Hepatic IR, Glucose, and Lipid Dysfunction

**FIGURE 3:**
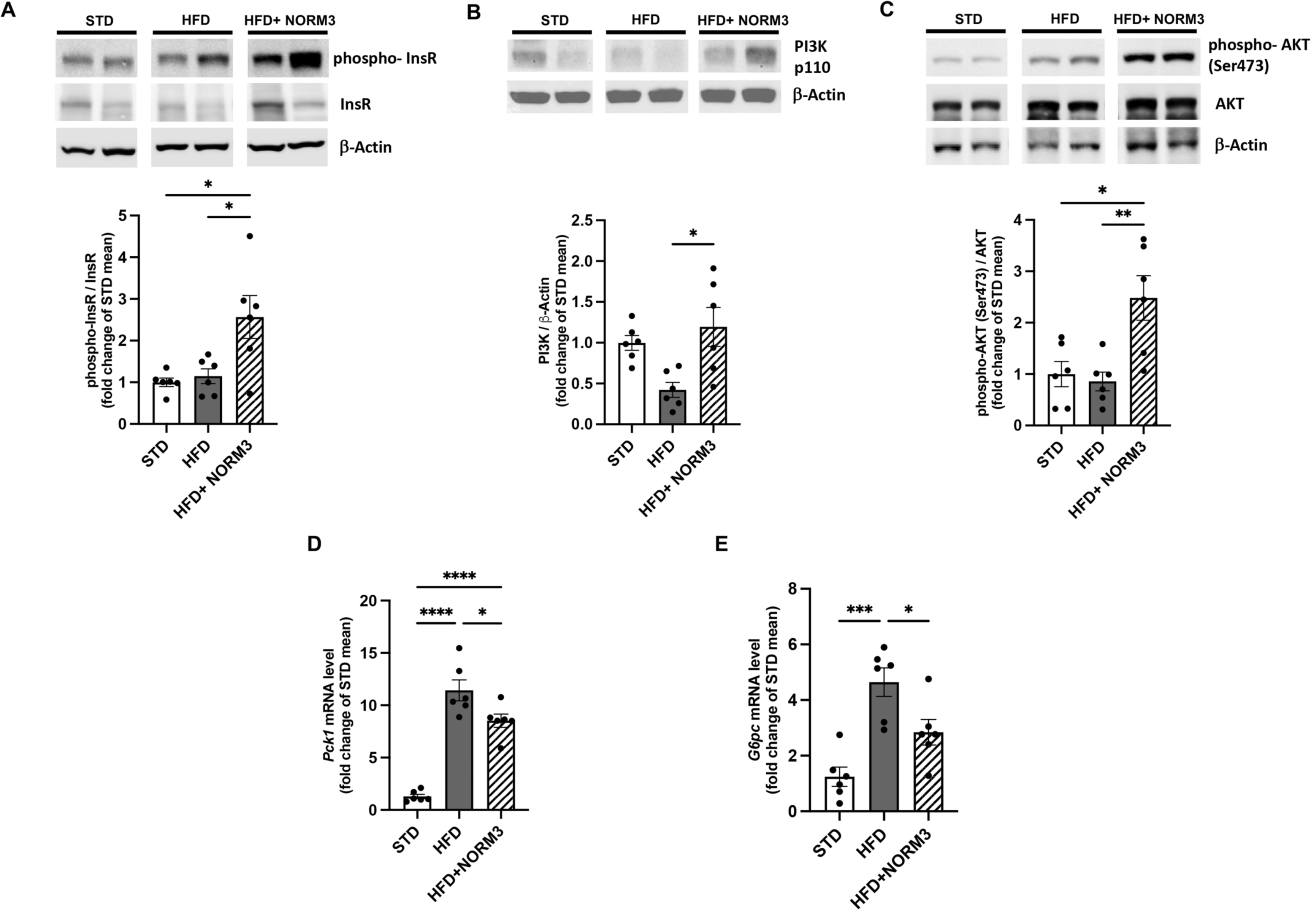
NORM3 improves hepatic insulin signaling and gluconeogenesis altered by HFD. Cropped images of the phospho‐InsR/InsR (A), PI3K (B), and phospho‐AK/AKT (C) were obtained by Western blot analysis in the livers of all animals (*n* = 6 for each group). The transcription of enzymes catalyzing hepatic gluconeogenesis *Pck1* (D) and *G6pc* (E) was measured by RT‐PCR (*n* = 6 for each group). Data are presented as mean ± SEM. *p < 0.05, **p < 0.01, ***p < 0.001, and ****p < 0.0001 versus STD or HFD.

**FIGURE 4:**
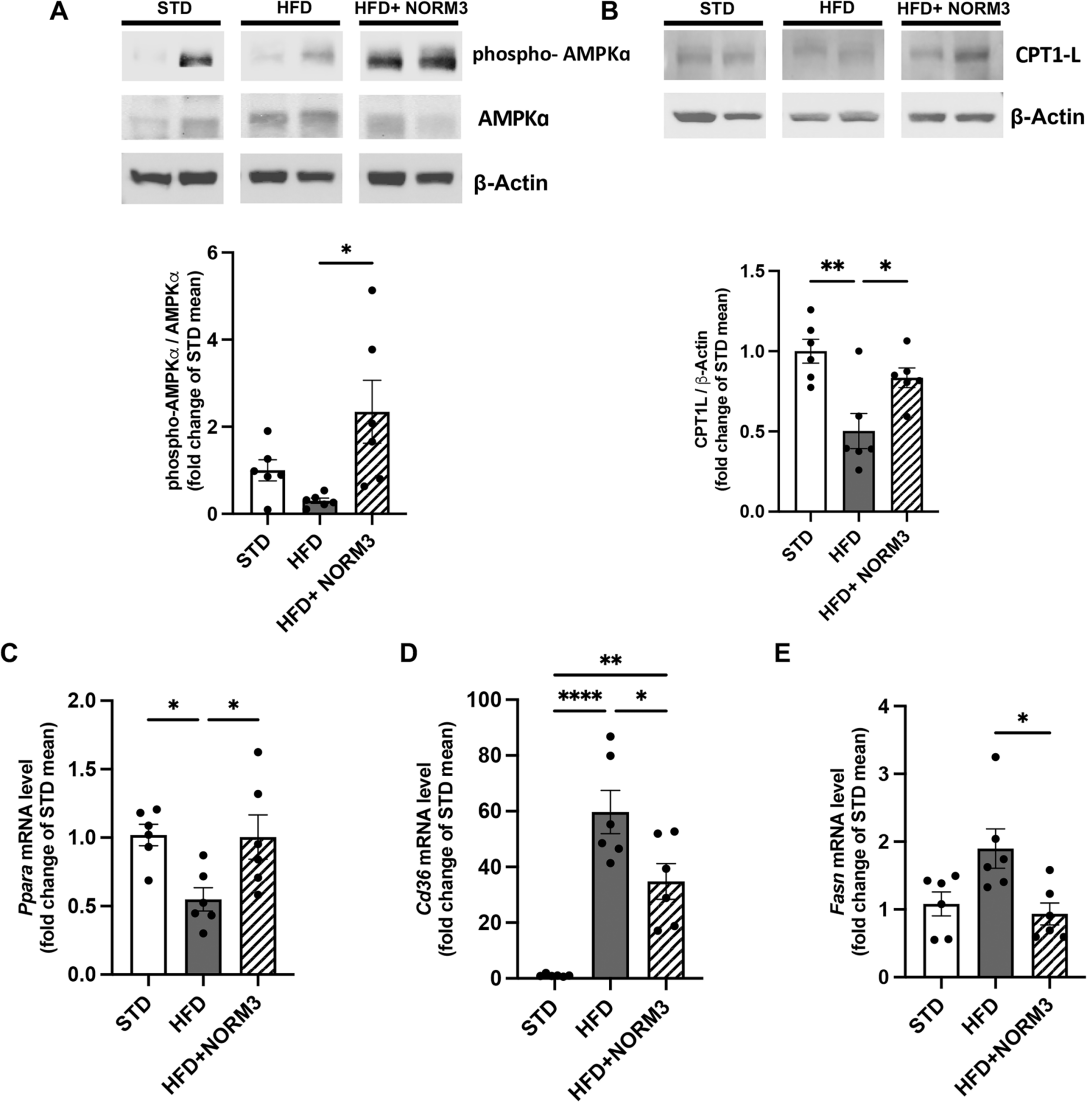
NORM3 dampens the alteration of lipid metabolism in the liver of HFD‐fed mice. Cropped images of Western blot for phospho‐AMPK/AMPK (A) and CPT1‐L (B) were shown (*n* = 6 for each group). The mRNA expression of *Ppara* (C), *Cd36* (D), and *Fasn* (E) was evaluated by RT‐PCR (*n* = 6 for each group). Data are presented as mean ± SEM. *p < 0.05, **p < 0.01, and ****p < 0.0001 versus STD or HFD.

Then, we explore the effect of NORM3 treatment on hepatic glucose homeostasis, demonstrating its capability to improve insulin signaling altered by HFD. Indeed, NORM3 increased the phosphorylation of insulin receptor (InsR) and the activation of the PI3K/AKT pathway (Figure 3A–C), compared with the HFD group. Moreover, NORM3 significantly decreased HFD‐altered transcription of the two primary enzymes that control key gluconeogenesis steps, *Pck1* and *G6pc* (Figure 3D,E). The effect of NORM3 on the hepatic lipid dysmetabolism induced by HFD was also investigated. Seven‐week administration of NORM3 significantly increased the phosphorylation of AMPK compared to the HFD group (Figure 4A); this kinase is a crucial energy sensor involved in glucose and lipid metabolism. NORM3 also restored the protein expression of CPT1 to STD levels (Figure 4B) and consistently increased HFD‐altered expression of *Ppara* (Figure 4C) and reduced *Cd36* and *Fasn*, both critical markers of steatosis (Figure 4D,E).

### 3.4 Effect of NORM3 on Liver Morphology and Inflammation of HFD‐Fed Mice

**FIGURE 5:**
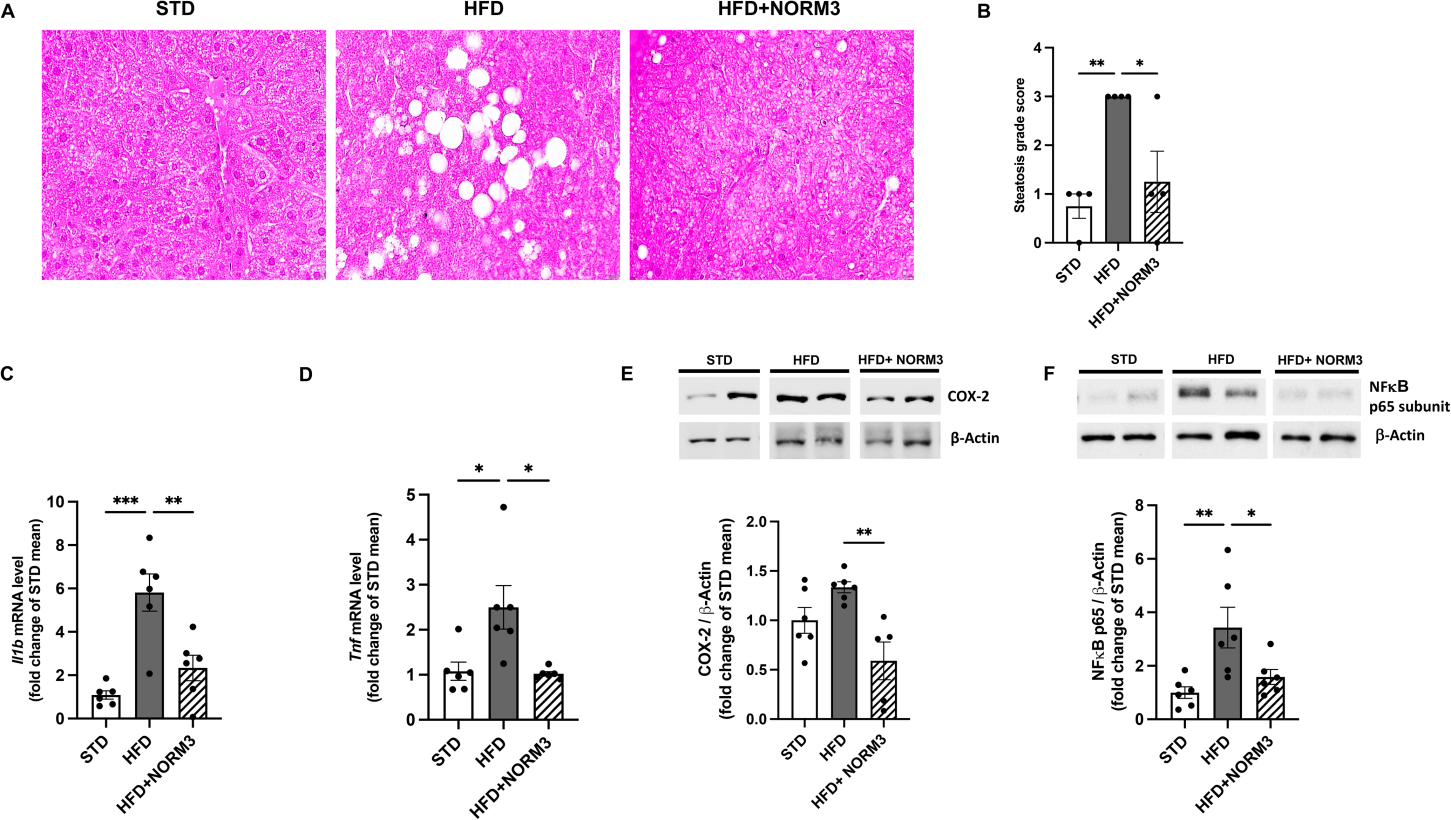
Effect of NORM3 on liver morphology and inflammation of obese mice. Representative paraffin‐embedded sections of the liver were stained with H&E (A), and the degree of steatosis (B) was examined in a double‐blinded manner (*n* = 4 for each group). Hepatic pro‐inflammatory *Il1b* (C) and *Tnf* (D) were evaluated by RT‐PCR (*n* = 6 each group); the protein expression of COX‐2 (E) and NFκB p65 subunit (F) was also assessed (*n* = 6 each group). Data are presented as mean ± SEM. *p < 0.05, **p < 0.01, ***p < 0.001 versus STD or HFD.

In HFD mice, liver morphological evaluations revealed moderate to severely degenerated hepatocytes with optically empty cytoplasm containing small and large, rounded vacuoles that delocalize the nucleus to the periphery, all features typical of micro‐ and macro‐vacuolar steatosis, compared with the STD liver (Figure 5A,B). Notably, NORM3 treatment counteracted hepatocyte morphological alteration induced by HFD. Based on the well‐known anti‐inflammatory activity of NORM3 components (PEA‐Rut and HT), we demonstrated the marked protective effect of their association in reducing the transcription of pro‐inflammatory mediators, such as IL‐1β and TNF‐α (Figure 5C,D), as well as the protein expression of COX‐2 enzyme and the nuclear translocation of p65 NFκB (Figure 5E,F), all parameters altered by HFD.

### 3.5 Antioxidant Activity of NORM3 in the Liver of Obese Mice and In Vitro Evidence for the Synergistic Effect of Its Single Components

**FIGURE 6:**
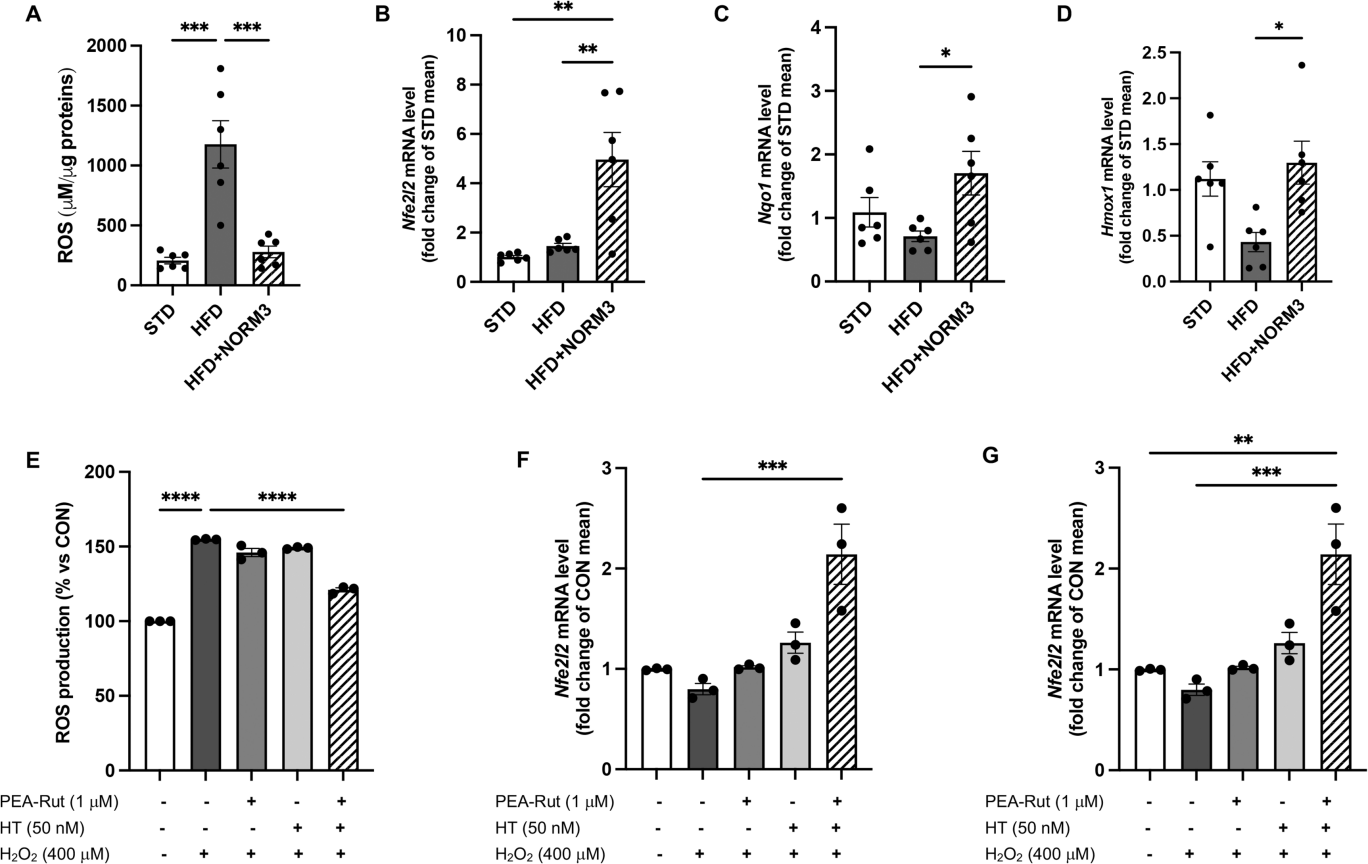
NORM3 counteracts hepatic oxidative stress in vivo and in vitro: synergistic effects of its single components. ROS production was evaluated by spectrofluorimetric analysis in both livers of obese mice (*n* = 6 each group) (A) and HepG2 cells treated with PEA‐Rut and HT (for 24 h), alone or in combination, and challenged with H_2_O_2_ (for 4 h) (E). Detoxifying gene expression of *Nfe2l2* (B, F), *Nqo1* (C), and *Hmox1* (D), as well as inflammatory *Il1b* (G), were also determined by RT‐PCR analysis (*n* = 6 each group). In vitro data are presented as means ± SEM from three different experiments in duplicate. *p < 0.05, **p < 0.01, ***p < 0.001, ****p < 0.0001 versus STD or HFD or versus untreated control cells.

NORM3 counteracted hepatic oxidative stress caused by HFD, reducing ROS production (Figure 6A) and stimulating the hepatic detoxifying system as shown by the increased mRNAs of antioxidant NRF2 (*Nfe2l2*), NQO1 (*Nqo1*), and HO1 (*Hmox1*) compared to HFD mice (Figure 6B–D).

To investigate the synergistic effect of NORM3 components, HepG2 cells were treated with PEA‐Rut and HT, alone or in combination, at the same rate and proportional concentrations used in the examined formulation in vivo. H_2_O_2_ challenge was used to mimic the detrimental conditions (oxidative stress and associated inflammation), characterizing liver damage related to IR. In our experimental conditions, these compounds, used alone or in combination, did not modify cell viability (data not shown). Notably, PEA‐Rut or HT did not arise any significant protective effect when used alone after the H_2_O_2_ challenge, while only the combination PEA‐Rut + HT was able to reduce cellular ROS amount (Figure 6E), to increase the mRNAs of detoxifying factor *Nfe2l2* and reduce the inflammatory cytokine *Il1b* (Figure 6F,G).

## 4 Discussion

Given the therapeutic drawbacks and side effects of drugs for treating diabesity and related NAFLD or MAFLD, the exploration of new therapeutic natural compounds is needed. Therefore, we investigated the effects of NORM3, a nutritional supplement containing co‐micronized PEA‐Rut associated with HT, in a mouse model of diabesity induced by HFD feeding. The beneficial effects of hepatic glucose and lipid dysmetabolism, inflammation, and oxidative stress are attributed to the pleiotropic mode‐of‐action of NORM3 due to its chemical composition, which is rich in different bioactive compounds.

As an insulin‐sensitive organ, the liver plays a pivotal role in regulating metabolic homeostasis. In response to metabolic dysfunctions, hepatic IR is one of the early‐developed pathological features, and improving IR has excellent potential for treating hyperglycemia and lipid storage in the liver (Norton et al. 2022). We report that the treatment of NORM3 significantly reduced body weight, fat mass, and IR in obese mice, conceivably due to the additive/synergistic effect of PEA‐Rut with HT. Indeed, our previous data showed that PEA alone, at a higher dose (30 mg/kg) and later in time, counteracts metabolic inflexibility and hepatic mitochondrial dysfunction induced in mice by HFD, acting as a critical sensitizer of the AMPK enzyme (Annunziata et al. 2020). PEA also improved adipose tissue plasticity by restoring lipid metabolism and promoting the white‐to‐brown conversion of adipocytes in obese mice (Annunziata et al. 2022). Hepatoprotective properties and antidiabetic effects of the flavonoid rutin (used in a wide range of doses 5–100 mg/kg) were previously identified (Ghorbani 2017; Tobar‐Delgado et al. 2023) and shown to be the result of PPAR‐α and antioxidant enzyme increased expression in hepatocytes (Liu et al. 2017). HT (10–20 mg/kg) limited chronic inflammation and IR and decreased hepatic steatosis by the reduction of endoplasmic reticulum stress (Wang et al. 2018), nitrosative and/or oxidative stress (Vlavcheski, Young, and Tsiani 2019), and intestinal barrier permeability (Pirozzi et al. 2016) in obese mice or rats.

The relevant outcome of this study was the demonstration that NORM3 can improve insulin sensitivity and glucose tolerance, an effect that is not entirely due to the observed reduction in body weight but also to the beneficial effects on glucose homeostasis. Here, we showed that NORM3 decreased HOMA‐IR in HFD‐fed mice. Interestingly, the increased glucagon secretion by HFD was also normalized by NORM3. It is known that increased glucagon secretion predicts the worsening of glucose tolerance, and paradoxical glucagon hypersecretion and hyperglycemia were seen in diabetes (Honzawa, Fujimoto, and Kitamura 2019).

Recently, the etiology and mechanisms of IR have been reviewed (James, Stockli, and Birnbaum 2021). Genetic and biochemical studies advocate a pivotal role for adipose tissue in the development of IR, conceivably by releasing lipids and other circulating factors, among which pro‐inflammatory cytokines (TNF‐α, MCP‐1, and IL‐6) promote IR in other insulin‐sensitive organs, including the liver. When storage ability is overwhelmed, autocrine and endocrine functions of adipose tissues are altered. The subsequent accumulation of ectopic fat leads to lipotoxicity, promoting low‐grade inflammation and IR in the liver (Gross et al. 2017). As recently reviewed, tissue‐specific IR can induce systemic IR (James, Stockli, and Birnbaum 2021). Indeed, several animal studies have supported the concept that metabolic or signaling alterations in one tissue can have a systemic impact and influence insulin action and its effects in other organs, as evidenced by observations in humans (Gancheva et al. 2018).

Chronic tissue inflammation, considered a key feature of diabesity, is observed in insulin‐target tissues, contributing to systemic metainflammation underlying the interplay between immunologic processes and metabolic dysfunctions (Rohm et al. 2022). In our experimental conditions, NORM3 significantly decreased serum inflammatory cytokines (TNF‐α, MCP‐1 IL‐6) and increased the anti‐inflammatory IL10. Interestingly, NORM3 also reduced resistin, a pro‐inflammatory adipokine that links obesity to diabetes (Tripathi et al. 2020). Resistin induces a pro‐inflammatory response involved in the development and advancement of hepatocellular carcinoma in individuals with diabetes (Abdalla 2023).

In diabesity, the inflammatory process perturbs the intracellular concentration of several intermediates, leading to defects in cell responsiveness to insulin. Such intermediates may cause IR by inhibiting one or more of the downstream components in the insulin signaling cascade (James, Stockli, and Birnbaum 2021). Among others, insulin function is regulated by the activation of the insulin receptor/PI3K/AKT signaling pathway and the modulation of redox balance (Lennicke and Cocheme 2021). In IR, a derailment in the PI3K/AKT/GLUT‐4 signaling occurs in several organs, including the liver (Gao et al. 2015; Huang et al. 2018). Literature data indicate that the phosphorylation of AKT is also involved in the translocation of intracellular vesicles containing glucose transporter proteins to the plasma membrane, facilitating glucose utilization by tissues (Gao et al. 2019; Manning and Cantley 2007). Here, NORM3 lessened glucose intolerance and IR, as shown by OGTT and ITT. Indeed, NORM3 increased the phosphorylation of insulin receptors and activated the PI3K/AKT pathway, improving hormone signaling in the liver of obese mice.

Another hallmark of IR is the inability to suppress hepatic glucose output, mainly due to a sustained elevation of gluconeogenesis (James, Stockli, and Birnbaum 2021). In this context, NORM3 downregulates hepatic gluconeogenesis, as shown via PTT and confirmed by the reduction of key gluconeogenic enzymes, phosphoenolpyruvate carboxykinase, and glucose‐6‐phosphatase.

NORM3 exerts its hepatoprotective effects, also improving serum lipid profile (triglycerides and cholesterol) and liver enzymes (transaminases and LDH). Indeed, all components of NORM3 had previously shown lipid‐lowering activities. For instance, HT suppressed the *de novo* synthesis and increased the catabolism of fatty acids (Cao et al. 2014; Pirozzi et al. 2016). Accordingly, HT administration showed several anti‐atherosclerotic effects, including the regulation of blood lipid profiles, the decrease of inflammatory mediators (Tejada et al. 2017), and the improvement of hepatic mitochondrial dysfunction induced by HFD (Ortiz et al. 2020). Our previous data demonstrated that in the same experimental model, PEA (30 mg/kg/*die* per os) increased liver AMPK phosphorylation, reduced lipid synthesis, and induced PPAR‐α expression (Annunziata et al. 2020). Rutin, as well as other phytochemicals, has been shown to improve diabetes in clinical trials not only by lowering blood glucose and reducing IR but also by improving lipid profile and inhibiting inflammation and oxidative stress (Bazyar et al. 2023; Luo et al. 2023).

Here, we show that NORM3 increased the phosphorylation of AMPK, a crucial sensor of cellular metabolism, and restored the expression of CPT 1, the first rate‐limiting step of β‐oxidation of long‐chain fatty acids. AMPK has been identified as a therapeutic target for metabolic disease treatment (Day, Ford, and Steinberg 2017), leading to the phosphorylation of key metabolic mediators and transcriptional regulators, including PPARs. Indeed, AMPK activation redirects metabolism toward inhibited anabolism and increased catabolism, limiting glucose and lipid synthesis and promoting fatty acid oxidation as an energy source (Herzig and Shaw 2018). Consistently, NORM3 markedly increased the expression of different genes involved in hepatic fatty acid oxidation (PPAR‐α and CPT1) and decreased lipid transport and *de novo* synthesis of fatty acid (CD36 and FAS, respectively), thus improving hepatic lipid metabolism.

The modulation of genes and proteins involved in hepatic glucose and lipid metabolism and steatosis by NORM3 contributes to the improvement of IR and the anti‐inflammatory effects. The role of cytokine‐ and enzyme‐induced inflammation and their link with IR and NAFLD has been defined (Duan et al. 2022). The increasing understanding of molecules and signaling pathways involved in diabesity‐driven inflammation allows the identification or development of novel preventive and therapeutic approaches to NAFLD or MAFLD (Song et al. 2023). Here, we show that NORM3 markedly reduced the expression of inflammatory cytokines (TNF‐α and IL‐6), COX‐2, and NFkB activation in the liver, confirming its anti‐inflammatory activity, likely associated with its ability to control metabolic alterations through PPAR‐α recovery.

Metabolic disarrangements, mitochondrial impairment, and oxidative stress have a crucial role in inducing hepatocyte damage and NAFLD progression (Sanches et al. 2023; Zheng et al. 2023). Lipid peroxidation products associated with inflammatory cytokines (i.e., TNF‐α) contribute to mitochondrial dysfunction, causing the enhancement of ROS production, which in turn self‐sustains organelle damage. Oxidative stress is commonly associated with an imbalance between intracellular ROS levels and endogenous enzymatic and nonenzymatic antioxidants (Ezhilarasan and Lakshmi 2022). NRF2 is a transcriptional factor that orchestrates the cellular defense mechanisms against oxidative stress induced by obesity and other redox insults (Vasileva et al. 2020). Excessive oxidative stress in the cell activates NRF2, which upregulates genes that encode major cytoprotective enzymes such as NQO1 and HO1. Recent studies showed that the activation of the NRF2/HO‐1 signaling pathway could attenuate NASH via ameliorating oxidative stress (Xu et al. 2018). Moreover, NRF2 knockout mice showed a more rapid NASH development than wild‐type ones on HFD (Okada et al. 2013). Several plant‐derived compounds are considered NRF2 activators and hypothetically achieved as cytoprotective compounds in metabolic stress status like diabesity (Vasileva et al. 2020).

Interestingly, NORM3 significantly induced the hepatic transcription of NRF2 and its downstream target genes HO‐1 and NQO1, indicating NRF2/HO‐1 detoxifying system activation. Notably, the activated AKT promotes NRF2 translocation to the nucleus and then the transactivation of the downstream target genes, inhibiting oxidative stress in HFD‐induced obesity (Li et al. 2020; Liu et al. 2016). Our results suggest the crucial role of AKT in NORM3 effects, consistently with the involvement of AKT activation in improving insulin sensitivity, regulation of gluconeogenesis, and recovery of hepatic oxidative redox balance in diabetes and obesity (Huang et al. 2018).

Finally, to investigate the synergistic effect of the components of NORM 3, we employed an in vitro model of H_2_O_2_‐induced oxidative stress using the hepatocyte cell line HepG2. These cells are sensitive to oxidative stress because NADPH oxidase (NOX)‐generated ROS and H_2_O_2_ challenge increased hepatocyte and DNA damage, causing cytotoxicity (Tu et al. 2022). Interestingly, we show that combining the compounds constituting NORM3 rather than the single components alone reduces the oxidative stress and inflammation induced by the H_2_O_2_ challenge in hepatocytes, which mimics in vitro one of the main detrimental features characterizing hepatic damage and IR.

## 5 Conclusions

Diabesity, that is, diabetes related to obesity, is a health problem that usually develops over many years and involves the interplay of multiple factors, including genetic, psychological, nutritional, hormonal, and immune aspects. Considering the multiple underlying mechanisms and their multifaceted interrelations, it is inappropriate to target a single player, and it would be naive to search for a unidirectional remedy. Finetuning of this complex pathological process could be achieved only by multitarget therapies with synergistic and converging effects in managing diabesity.

Here, our data show that the association of natural compounds with anti‐inflammatory and metabolic effects, namely NORMAST 3, can restore hepatic glucose and lipid metabolism, improve local and systemic insulin sensitivity, limit inflammation and oxidative stress, and promote antioxidant redox balance. Further studies will be needed to evaluate the preventive efficacy of NORMAST 3 supplementation to counteract the onset of diabesity based on the synergistic effect of all its components.

## Author Contributions

**S. Melini:** data curation, investigation, methodology, writing – original draft. **C. Pirozzi:** conceptualization, data curation, investigation, writing – original draft, writing – review and editing. **A. Lama:** data curation, investigation, visualization. **F. Comella:** investigation, visualization. **N. Opallo:** investigation. **F. Del Piano:** investigation. **E. Di Napoli:** investigation. **M. P. Mollica:** data curation, investigation. **O. Paciello:** data curation, investigation. **M. C. Ferrante:** validation, visualization. **G. Mattace Raso:** conceptualization, data curation, supervision, validation, writing – review and editing. **R. Meli:** conceptualization, data curation, supervision, validation, writing – original draft, writing – review and editing.

## Conflicts of Interest

The authors declare no conflicts of interest.

## Supporting information


Figure S1.

## Data Availability

The data that support the findings of this study are available from the corresponding author upon reasonable request.
