# Supplementary material for: Co‐Micronized Palmitoylethanolamide and Rutin Associated With Hydroxytyrosol Recover Diabesity‐Induced Hepatic Dysfunction in Mice: In Vitro Insights Into the Synergistic Effect
Source: Phytother Res. 2024 Oct 30;38(12):6035–47. doi: 10.1002/ptr.8361 (PMC11634826; doi:10.1002/ptr.8361)

Original Western blots

Phospho-IR/IR

Blot n.1

250 kDa
150 kDa
95 kDa →
75 kDa

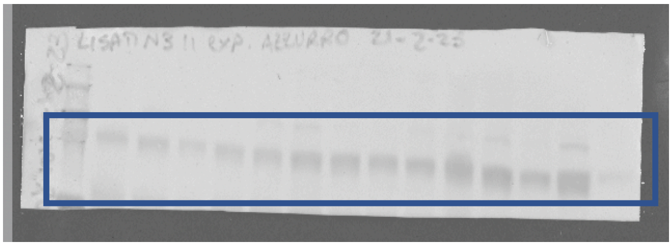

→ **Phospho-IR**

250 kDa
150 kDa
95 kDa →
75 kDa

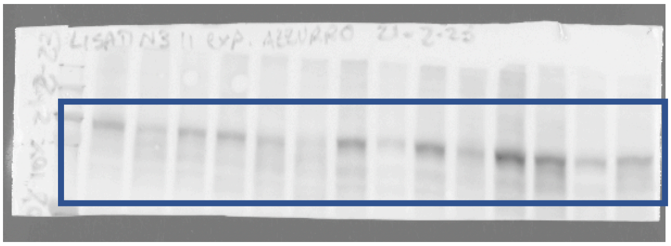

→ **IR**

Blot n.2

250 kDa
150 kDa
95 kDa →
75 kDa

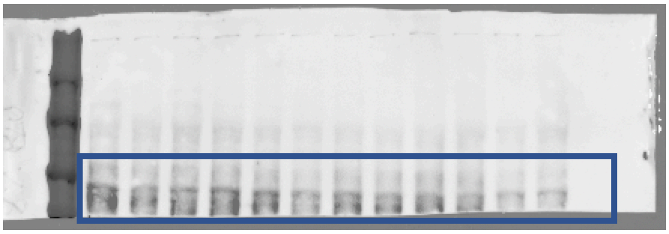

→ **Phospho-IR**

250 kDa
150 kDa
95 kDa →
75 kDa

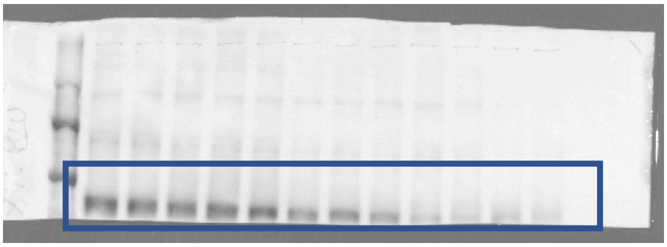

→ **IR**

Phospho-AKT/AKT

Blot n.1

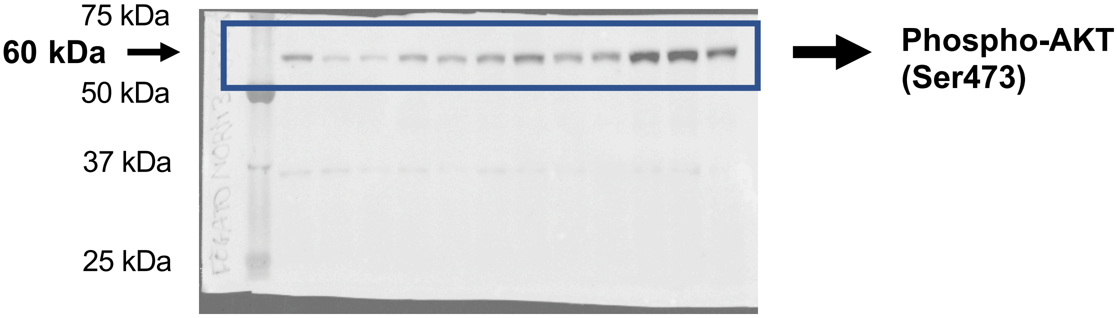

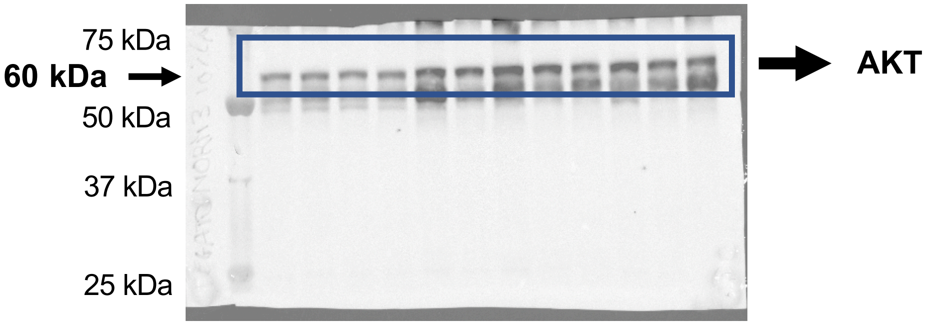

Blot n.2

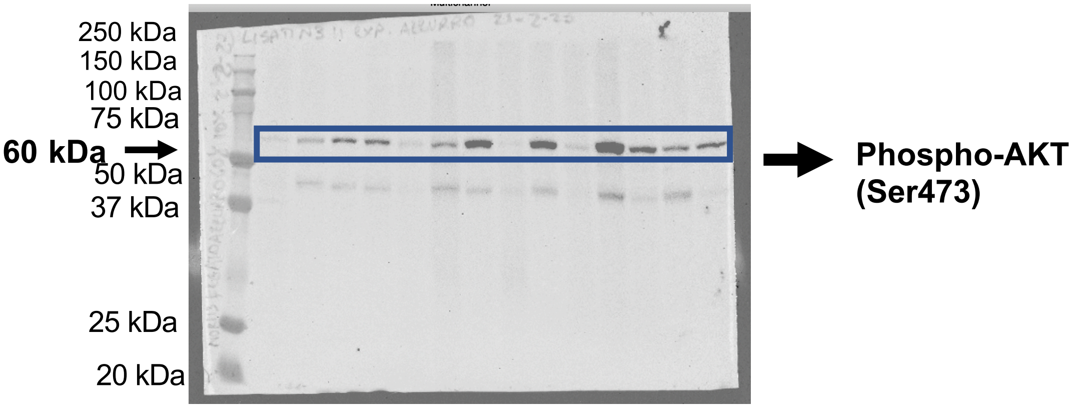

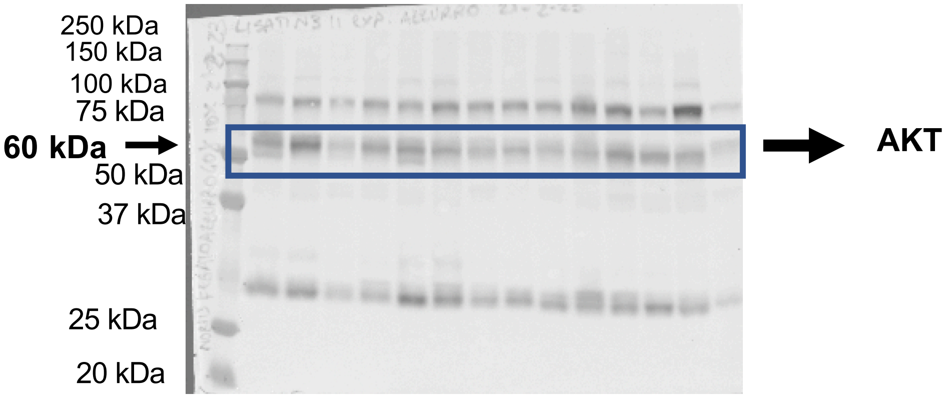

PI3K/β-Actin

Blot n.1

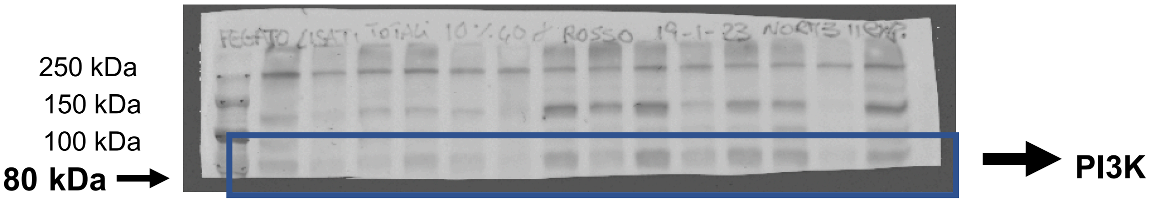

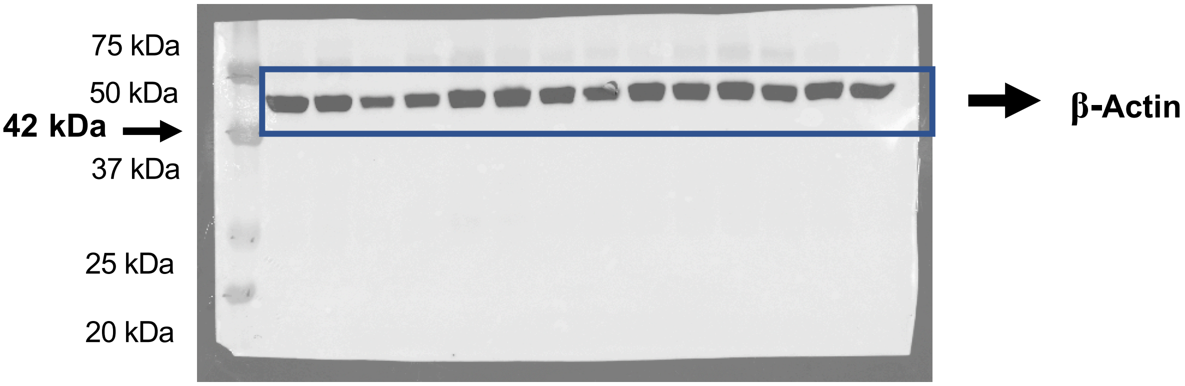

Blot n.2

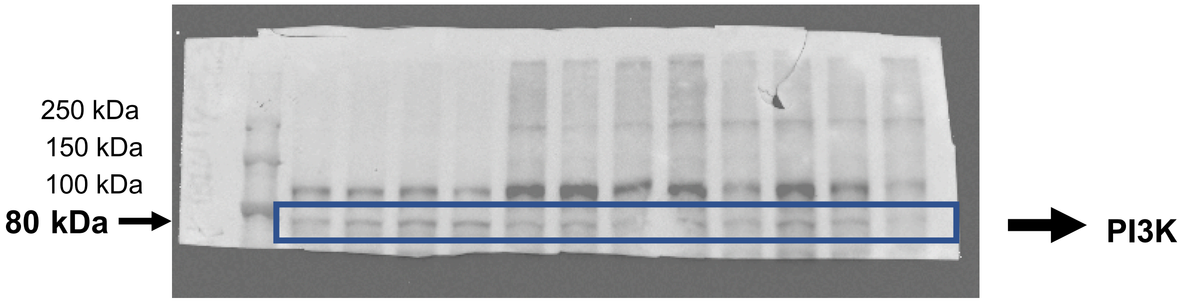

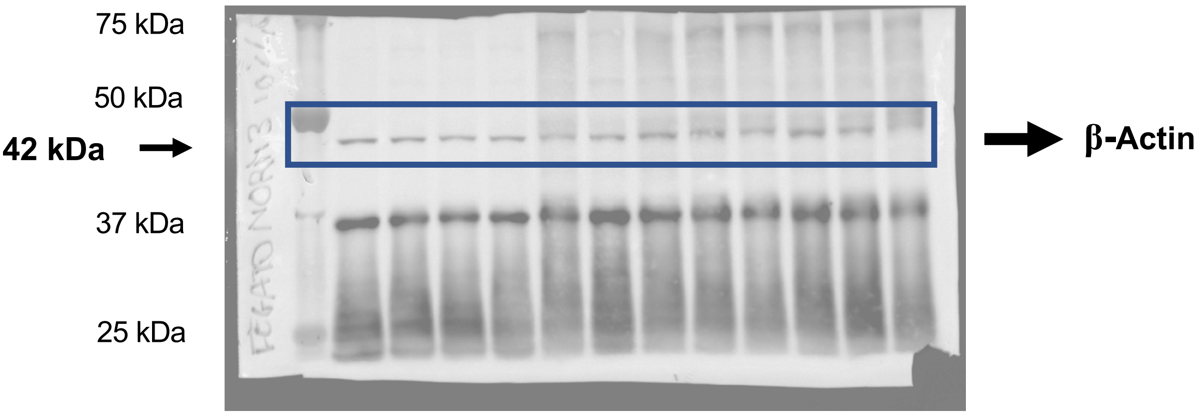

Phospho-AMPK/AMPK

Blot n.1

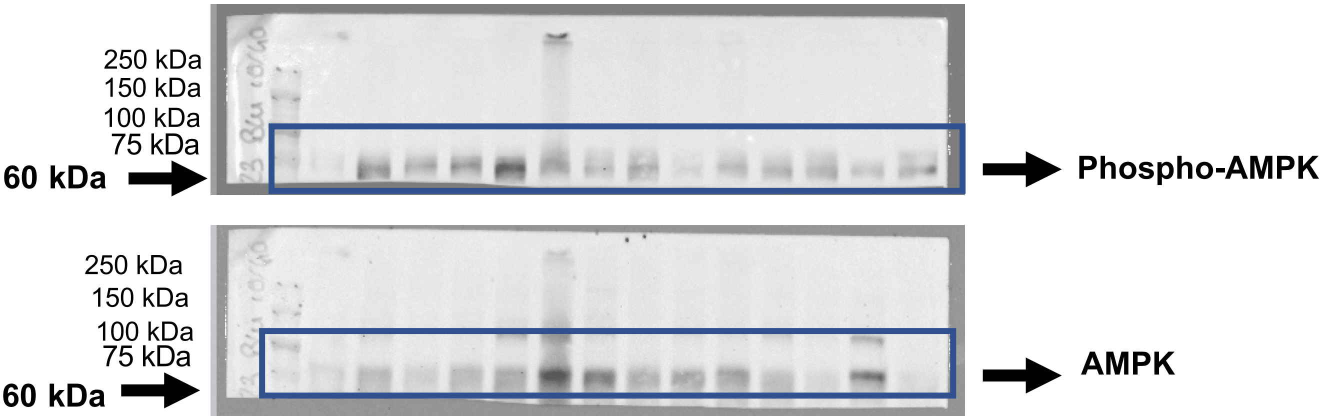

Blot n.2

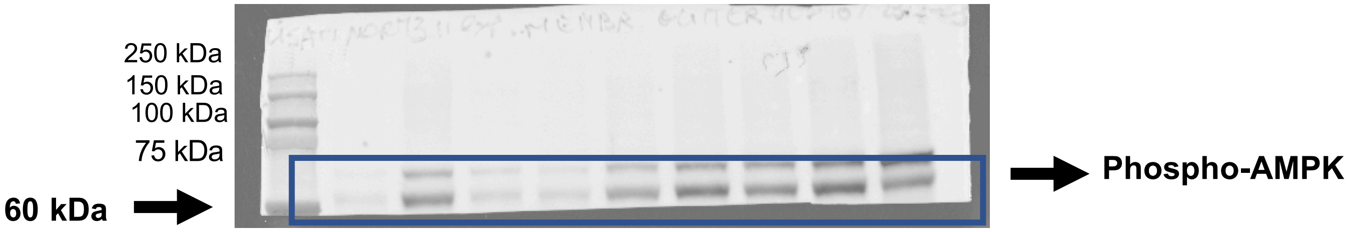

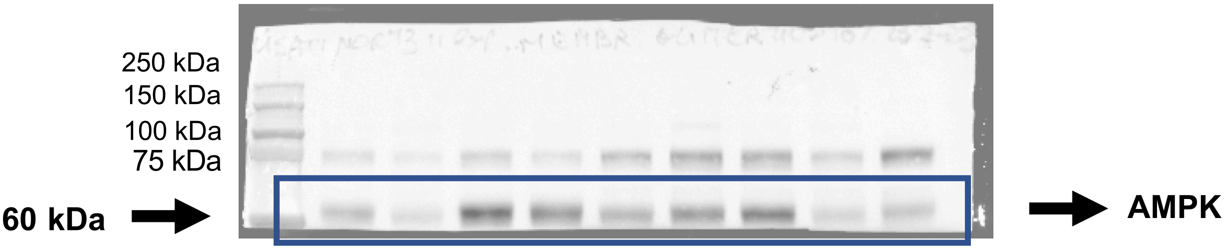

CPT-1L/β-Actin

Blot n.1

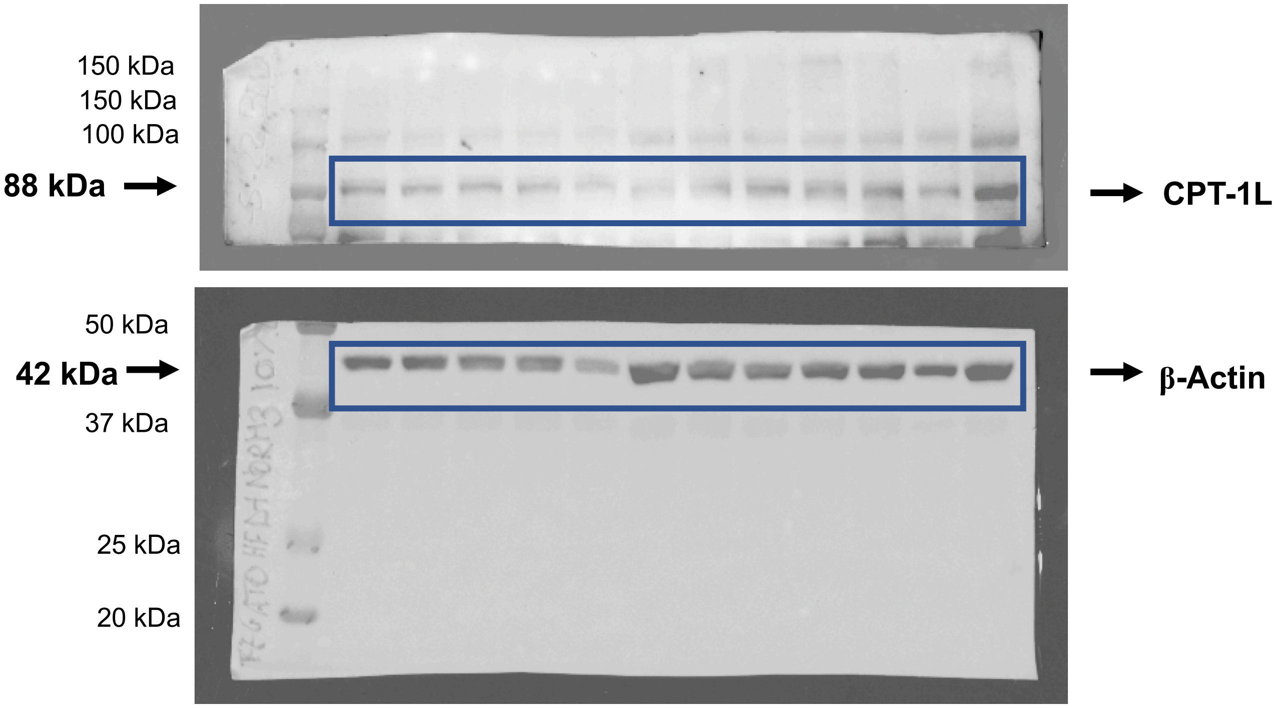

Blot n.2

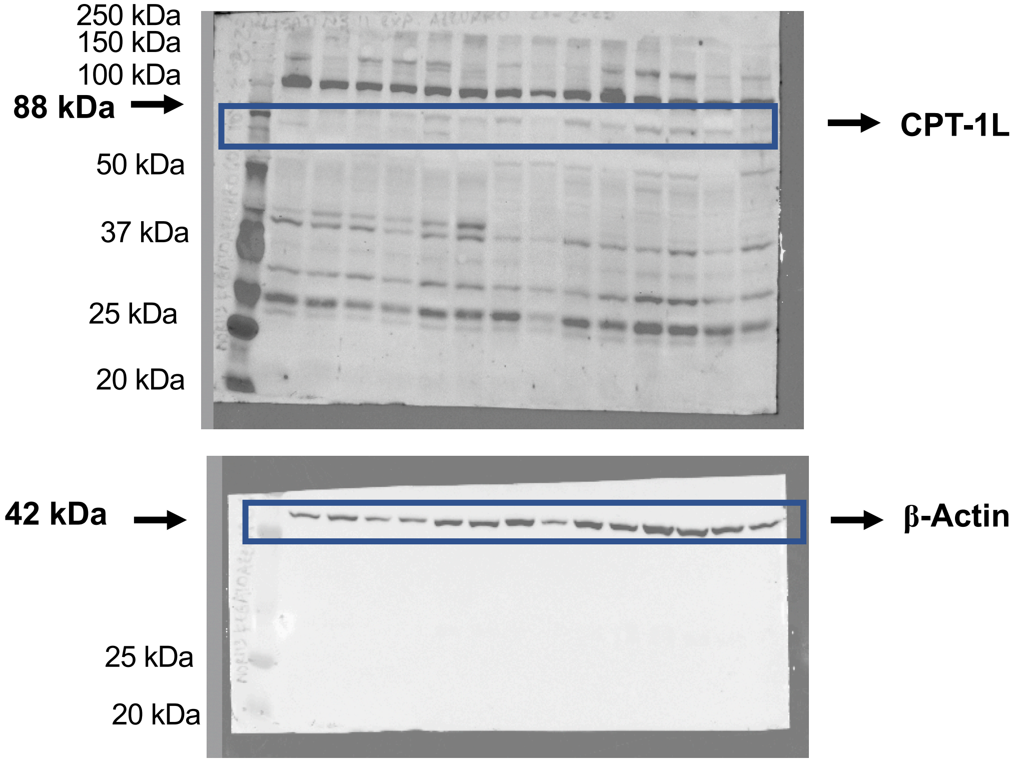

NF-κB/β-Actin

Blot n.1

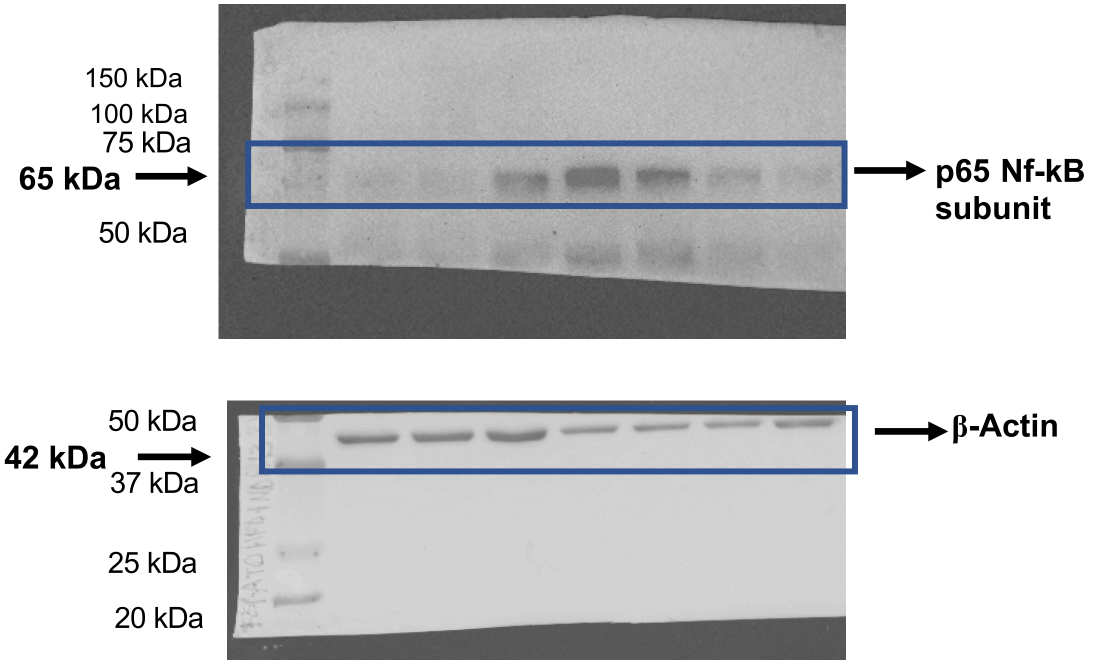

Blot n.2

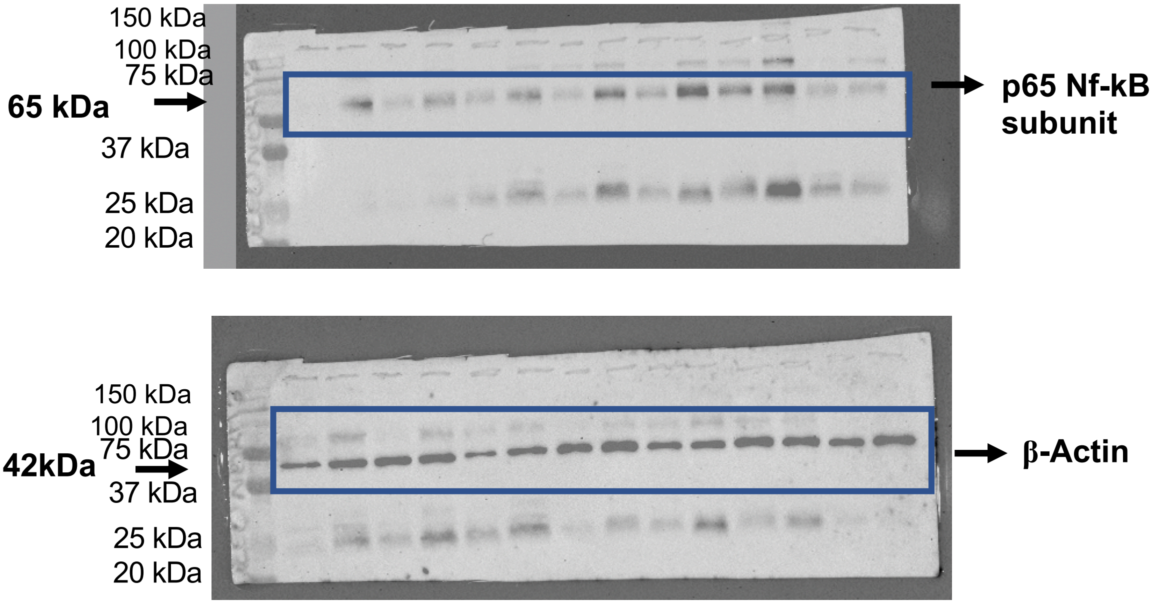

COX2/β-actin

Blot n.1

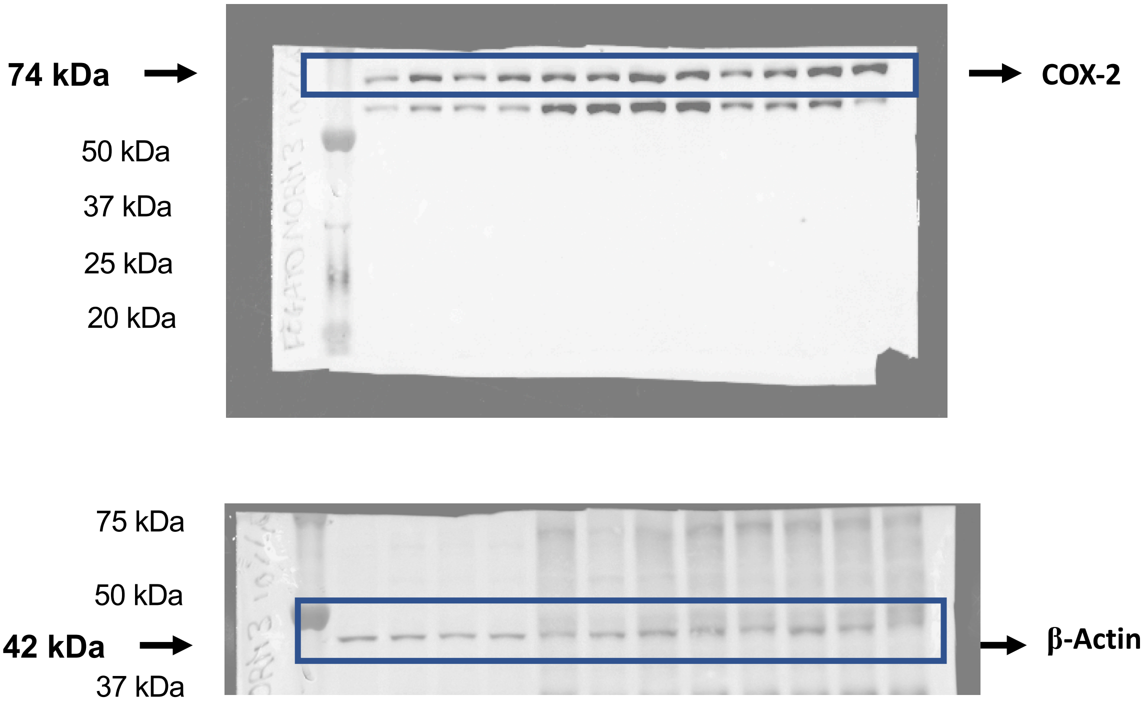

Blot n.2

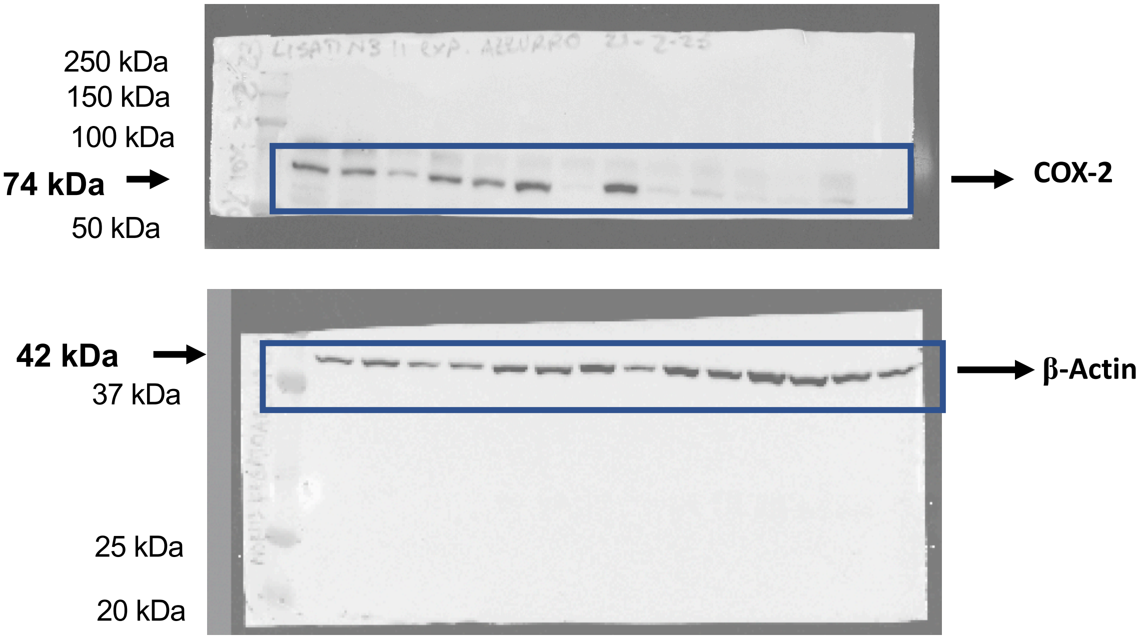

Supplement: Supplementary file 1 — Figure S1. [file PTR-38-6035-s001.pdf]
